# The Effects of Wenxin Keli on Left Ventricular Ejection Fraction and Brain Natriuretic Peptide in Patients with Heart Failure: A Meta-Analysis of Randomized Controlled Trials

**DOI:** 10.1155/2014/242589

**Published:** 2014-04-27

**Authors:** Yu Chen, Xingjiang Xiong, Chunmei Wang, Chenggang Wang, Ying Zhang, Xingyong Zhang, Yonghong Gao, Yanhui Xing, Jun Li, Jie Wang, Xiaoqiu Liu, Yanwei Xing

**Affiliations:** ^1^Shenyang Pharmaceutical University, Shenyang, Liaoning 110016, China; ^2^Guang'anmen Hospital, Chinese Academy of Chinese Medical Sciences, Beijing 100053, China; ^3^Beijing An Zhen Hospital of the Capital University of Medical Sciences, Beijing 100029, China; ^4^Beijing XUANWU Traditional Chinese Medicine Hospital, Beijing 100050, China; ^5^The Key Laboratory of Chinese Internal Medicine of the Ministry of Education, Dongzhimen Hospital Affiliated to Beijing University of Chinese Medicine, Beijing 100700, China; ^6^Institute of Information on Traditional Chinese Medicine, China Academy of Chinese Medical Sciences, Beijing 100700, China

## Abstract

*Objective*. To evaluate the beneficial and adverse effects of Wenxin Keli (WXKL), either alone or in combination with Western medicine, on the left ventricular ejection fraction (LVEF) and plasma brain natriuretic peptide (BNP) in the treatment of heart failure (HF). *Methods*. Seven major electronic databases were searched to retrieve potential randomized controlled trials (RCTs) designed to evaluate the clinical effectiveness of WXKL, either alone or in combination with Western medicine, for HF, with the LVEF or BNP after eight weeks of treatment as main outcome measures. The methodological quality of the included studies was assessed using criteria from the Cochrane Handbook for Systematic Review of Interventions, Version 5.1.0, and analyzed using RevMan 5.1.0 software. *Results*. Eleven RCTs of WXKL were included. The methodological quality of the trials was generally evaluated as low. The risk of bias was high. The results of the meta-analysis showed that WXKL, either alone or in combination with Western medicine, was more effective in LVEF and BNP, compared with no medicine or Western medicine alone, in patients with HF or HF complicated by other diseases. Five of the trials reported adverse events, while the others did not mention them, indicating that the safety of WXKL remains uncertain. *Conclusions*. WXKL, either alone or in combination with Western medicine, appears to be more effective in improving the LVEF and BNP in patients with HF and HF complications.

## 1. Introduction

Chronic heart failure (HF) is a serious disease that endangers human life. A sudden fatal ventricular arrhythmia is usually the cause of death. A clinical trial indicated that the long-term mortality for patients hospitalized with HF improved from 1991 to 1997, but the mortality remains very high [1]. Porcine brain natriuretic peptide (pBNP) was first isolated from acid extracts of the porcine brain [2]. In recent years, plasma brain natriuretic peptide (BNP) levels have been used increasingly to evaluate the prognostic impact of a therapeutic strategy. The result was mainly obtained through an increase in ACEI and beta-blocker dosages. In optimally treated congestive heart failure (CHF) patients, the BNP-guided strategy reduced the risk of CHF-related death or hospital stays for CHF [3]. The BNP level is a good indicator for evaluating the improvement of HF because there is a good correlation between the BNP level and the severity of HF. The mortality risk increased at rising BNP and NT-proBNP levels [4].

Antiarrhythmic drug therapy for HF is the preferred option, but the curative effect of pure Western medicine on HF is unsatisfactory. Amiodarone is one of the most widely used antiarrhythmic drugs in clinical applications. Along with its therapeutic effect, amiodarone also has serious side effects [5]. It is particularly necessary to explore traditional Chinese medicine for the treatment of HF and to give full consideration to the role of Chinese medicine treatment in HF treatment.

Wenxin Keli (WXKL) is a type of traditional Chinese medicine developed by Guang'anmen Hospital, Chinese Academy of Chinese Medical Sciences. It is composed of five main components:* Nardostachys chinensis Batal* extract,* Codonopsis*, notoginseng, amber, and* rhizoma polygonati*. WXKL has been used to treat CHF and arrhythmias. Modern research has proven that WXKL can improve the cardiac myocytes' electrophysiological destabilization and cell structure of chronic heart failure. WXKL has a significant effect on controlling a variety of arrhythmias, right ventricular contractions, atrial premature contractions, atrial fibrillation (AF), and sinus tachycardia. It is safe, reliable, and appropriate for long-term use [6].

Currently, WXKL combined with antiarrhythmic drugs, a new integrative medicine therapy, has been widely used as an alternative and effective method for HF in China. A large number of clinical studies reported the clinical effect of WXKL and WXKL combined with antiarrhythmic drugs. And, until now, a large number of randomized controlled trials (RCTs) and case series have been published but have not been evaluated according to the Preferred Reporting Items for Systematic Reviews and Meta-Analyses (PRISMA) systematic review standard. And the predictive value of the LVEF and BNP in HF patients treated with WXKL has not been determined. Understanding the effect of WXKL on LVEF and BNP could be valuable for the management of HF. Therefore, this study aims to assess the current clinical evidence of WXKL combined with antiarrhythmic drugs for HF and seeks to identify the relationship between BNP and HF in patients treated with WXKL and to evaluate the effectiveness and safety of WXKL for the treatment of HF.

## 2. Materials and Methods

### 2.1. Database and Search Strategies

Seven major electronic databases, including Chinese National Knowledge Infrastructure (CNKI), the Chinese Biomedical Literature Database (CBMdisc), Wanfang Database, the Chinese Scientific Journal Database (VIP), PubMed, EMbase, and the Cochrane Library, were searched to retrieve potential RCTs. The search included articles published up to July 2013. Other relative research papers were searched manually. The following search terms were used individually or in combination: “Wenxin Keli,” “Wenxinkeli,” “Wenxin Granule,” “Wenxin Granules,” “heart failure,” and “randomized controlled trial.” The bibliographies of the included studies were searched for additional references.

### 2.2. Inclusion and Exclusion Criteria

All the randomized controlled trials (RCTs) of patients with HF that studied prescriptions based on WXKL alone or combined with Western medicine compared with no medicine or Western medicine alone were included. There were no restrictions on language, population characteristics, or publication type. The primary outcome measure was the LVEF or BNP after eight weeks of treatment with WXKL, and the secondary outcome measure was adverse drug reactions (ADR). In duplicated publications of an identical clinical trial, the publication with more complete data and information was included.

### 2.3. Data Extraction and Quality Assessment

Two authors independently conducted the literature search, literature screening, and data extraction. The extracted data included the title of the study, authors, year of publication, article source, total number of cases, grouping, diagnosis standards, details of methodological information and treatment process, and the details of the control interventions, outcomes, and adverse effects for each study. Disagreements were resolved by discussion, and a consensus was reached through a third party. The methodological quality of the included trials was assessed according to the Cochrane Handbook for Systematic Review of Interventions, Version 5.1.0 [7], to address the following seven criteria: random sequence generation (selection bias), allocation concealment (selection bias), blinding of participants and personnel (performance bias), blinding of outcome assessments (detection bias), incomplete outcome data (attrition bias), selective outcome reporting (reporting bias), and other sources of bias. In addition, there are other sources of bias that are relevant only in certain circumstances. The quality of all the included trials was categorized as a low, unclear, or high risk of bias (“Yes” indicates a low risk of bias, “No” indicates a high risk of bias, and “Unclear” is otherwise). The included trials were sorted into three categories: low risk of bias (all of the criteria were rated as having a low risk of bias), unclear risk of bias (at least one item was unclear), or high risk of bias (at least one item was at a high risk of bias).

### 2.4. Data Synthesis

RevMan 5.1.0 software, provided by the Cochrane Collaboration, was used for the data analysis. Dichotomous data were expressed as the risk ratio (RR), and continuous outcomes were presented as the weighted mean difference (WMD) or standardized mean difference (SMD), and 95% confidence intervals (95% CI) were calculated for both types of data. A meta-analysis was performed if the intervention, control, and outcomes were the same or similar. The statistical heterogeneity was presented as significant when the *I* squared (*I* ^2^) value exceeded 50% or *P* < 0.1. In the absence of significant heterogeneity, we pooled the data using the fixed effects model (*I* ^2^ < 50%). If there was significant heterogeneity, we used the random effects model (*I* ^2^ > 50%) [7]. Publication bias would be evaluated using funnel plot analyses if a sufficient number of studies were found.

## 3. Results

### 3.1. Description of the Included Trials

**Figure 1:**
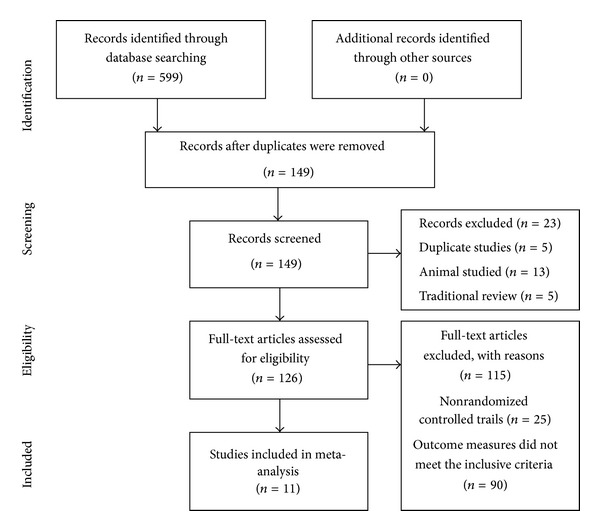
Flow chart of articles selection process.

After the primary search of the seven databases both in Chinese and English, 599 articles were retrieved: Cochrane Library (*n* = 0), PubMed (*n* = 3), Embase (*n* = 4), CNKI (*n* = 159), VIP (*n* = 120), CBMdisc (*n* = 156), and Wanfang (*n* = 157). The majority of these trials were excluded because some papers were found in more than one database and some included irrelevant titles and abstracts. Only 149 studies were retrieved. Following a review of the titles and abstracts, several studies were excluded, and only 126 studies remained. Five trials were excluded because of duplicated publications. Thirteen trials were excluded for being animal studies, and five trials were excluded for being nonclinical trials, including case reports and traditional reviews. One hundred and fifteen out of the remaining 126 articles were excluded based on the inclusion criteria, leaving eleven RCTs to be reviewed [8–18]. The screening process is summarized in a flow chart (Figure 1). All of the trials were conducted in China and published in Chinese. The characteristics of the eleven RCTs are summarized in Table 1.

The eleven RCTs involved a total of 907 patients with HF. Only five trials [10, 13, 16–18] specified the diagnostic criteria of HF. Of those, three [13, 16, 18] used the Chinese diagnosis and treatment of CHF guidelines (2007). One [10] used the guiding principle of the traditional Chinese medicine (TCM) treatment of CHF (1993). Another [17] used the ACC/AHA guidelines for the management of patients with HF without detailed information. The rest of the trials [8, 9, 11, 12, 14, 15] described patients with HF diagnoses by diagnostic criteria for CHF without detailed information.

The interventions of all eleven trials [8–18] included WXKL, either alone or combined with Western medicine, as shown in Table 1. The controls included Western medicine alone or no medicine use. The total treatment duration was eight weeks. Four trials [10, 13, 15, 17] specified the clinical standards of HF. All eleven trials [8–18] used the LVEF as the main outcome measure, and seven of the trials [12–18] used BNP as the main outcome measure. Five of the included trials [8, 12, 13, 16, 17] described the adverse effects in detail.

### 3.2. Methodological Quality of the Included Trials

The majority of the included RCTs were assessed as having low methodological quality. According to the predefined quality assessment criteria indicated above, none of the included trials were evaluated as having a low risk of bias, as is shown in Table 2. Only one [10] of the trials reported the methodology used to generate the allocation sequence. The trial stated the method was a table of random numbers method but did not provide detailed information; insufficient information was provided to allow for the quality assessment of the allocation method. Allocation concealment was not mentioned in all the included trials. The blinding of the participants, personnel, and outcome assessments was not detailed in all of the trials. Two trials [11, 13] reported dropouts or withdrawals. None of the trials calculated an estimation of the pretrial sample size, which indicated a lack of statistical power to ensure an appropriate estimation of the therapeutic effect. The results of the assessment of the risk of bias are presented in Table 2.

### 3.3. Effects of the Interventions

#### 3.3.1. Left Ventricular Ejection Fraction

**Figure 2:**
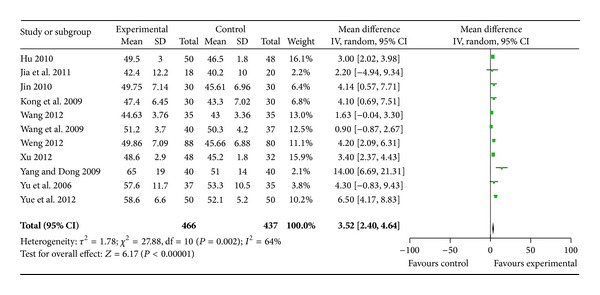
Analysis of left ventricular ejection fraction after eight months of treatment.

All the trials [8–18] used the improvement in the LVEF at eight months following treatment as an outcome measure. No significant difference in the LVEF before treatment was observed between the WXKL, either alone or combined with Western medicine, group (experimental group) and the Western medicine group (control group). This allowed for a comparison of the LVEF values of the two groups after treatment. The trial results for the eleven independent trials were not homogeneous, *X* ^2^ = 27.88, df = 10, (*P* = 0.002); *I* ^2^ = 64%, which required the use of the random effects model for the statistical analysis. The LVEF after WXKL treatment, either alone or combined with Western medicine, was higher than after Western medicine treatment alone. The meta-analysis demonstrated a significant difference between the two groups for each of the three criteria outcome measures (MD = 3.52, 95% CI = 2.40 to 4.64) (Figure 2). The main source of the heterogeneity may have several aspects. Firstly, the dosage and the time of WXKL the patients used were consistent. There are differences in the severity of HF of patients in the included trials. Secondly, for most of the eleven studies, the sample sizes were small. Thirdly, there are differences in the doctor's technology level. In addition, the diagnostic equipment of different hospital can make the results vary. So the clinical and methodological source made the heterogeneity of statistics.

#### 3.3.2. Brain Natriuretic Peptide

**Figure 3:**
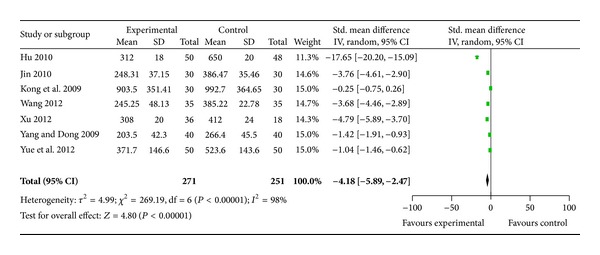
Analysis of brain natriuretic peptide after eight months of treatment.

Seven trials [12–18] used a reduction of BNP at eight months following treatment as an outcome measure. These trials compared the combination of WXKL with no medicine. The trial results for the seven independent trials were not homogeneous, *X* ^2^ = 269.19, df = 6, (*P* < 0.00001); *I* ^2^ = 98%, which required the use of the random effects model for the statistical analysis. The BNP level in the WXKL group (experimental group) was lower than that of the control group. The meta-analysis showed that there was a significant beneficial effect on the WXKL group compared with the control group, and there was a significant difference between the two groups for each of the three criteria outcome measures (SMD = −4.18, 95% CI = −5.89 to −2.47) (Figure 3). The main source of the significant heterogeneity of included trials may have several aspects. Firstly, the majority of the trials had poor methodological quality and a small sample size. Sample size calculation should be conducted before enrollment. If methodologically poorly designed, all the trials would show larger differences between experimental and control groups than those conducted rigorously. Secondly, there are differences in the severity of HF of patients in the included trials. Thirdly, in these trials, the method and instruments for testing the BNP level were various. This might lead to a significant heterogeneity of included trials. In addition, different diagnosis standards and different doctors' diagnosis can make a big difference. The diagnostic criteria of heart failure need to be unified and standardized in further research. So the clinical and methodological source made the heterogeneity of statistics.

### 3.4. Sensitivity Analysis, Subgroup Analysis, and Publication Bias

To examine the stability of the results, the fixed effects model was used to perform a meta-analysis of the LVEF and BNP instead of the random effects model. A significant difference was observed in the LVEF of the two groups for the three criteria outcome measures (MD = 3.17, 95% CI = 2.62 to 3.72). There was a significant difference between the WXKL group and the control group in the BNP levels (SMD = −1.73, 95% CI = −1.96 to −1.49). Given that the results of the two methods were consistent, the stability was considered to be sufficient. The number of trials was too small to conduct analyses of the subgroup and publication bias.

### 3.5. Adverse Effects

Five of the included trials [8, 12, 13, 16, 17] described adverse effects in detail, while the others did not mention adverse events. One trial [8] mentioned adverse effects in both groups, with four cases of dysphoria in the WXKL combined with the metoprolol group and six cases of worsening heart failure and three cases of a lowering of the blood pressure in the metoprolol group. None of the adverse events were serious. These side effects may be related to the adverse effect of metoprolol. One trial [12] reported that before and after treatment the routine blood studies, routine urine studies, liver function, and kidney function of patients in the two groups had no obvious changes, and no significant side effects were found after treatment. Another trial [13] mentioned that before and after treatment the liver function, kidney function, thyroid function, and chest X-ray films of patients in the treatment group did not change significantly. There was one case of dizziness, two cases of mild nausea, and one case of slight dry mouth in the treatment group. These symptoms are self-limited and did not affect the treatment. During the treatment period, there was one case of death due to exacerbations of HF in the control group. Two trials [16, 17] reported that all patients in the observation period experienced no significant ADR. The incidence of adverse reactions was lower in the treatment group compared with the control group.

## 4. Discussion

This systematic review included eleven RCTs with a total of 907 participants. The main findings of this review were that WXKL combined with Western medicine demonstrated a potential effect of improving the LVEF and lowering the BNP compared with Western medicine alone or no treatment. WXKL is an effective treatment for patients with HF. Because of the poor methodological quality of the included studies, the evidence for WXKL remains weak. The available data are not adequate to draw a definite conclusion regarding the efficacy of WXKL for HF, but they provide some encouraging evidence of the effect of WXKL for the treatment of HF. All the trials used the improvement in the LVEF at eight months following treatment as an outcome measure and trial results for the independent trials were not homogeneous. Seven trials used a reduction of BNP at eight months following treatment as an outcome measure and trial results were also not homogeneous. The main source of the heterogeneity may have several aspects. The dosage and the time of WXKL the patients used were consistent. Because of different diagnosis standards, different doctors' diagnosis can make a big difference. So the clinical and methodological source made the heterogeneity of statistics.

HF is one of the most common chronic arrhythmia conditions associated with an adverse prognosis and patients with HF have an increased risk of death. The effective treatment and prevention of HF has important clinical significance. BNP, also called B-type natriuretic peptide, is a member of the natriuretic peptides. Findings have suggested that BNP may be a useful screen for patients with left ventricular dysfunction. The mean BNP concentration was significantly greater in patients experiencing end points than in patients who had successful treatment of CHF [19]. Current studies show that the measurement of BNP plasma concentrations is a useful tool in the diagnosis of acute heart failure. BNP constitutes a promising new marker of prognosis after an acute coronary syndrome episode and in patients with CHF [20].

The curative effect of pure Western medicine on HF is unsatisfactory. During long-term Western medicine treatment, typical side effects build up [21]. Most of the pharmacologic therapies for HF are unsuccessful, so it is particularly necessary to explore traditional Chinese medicine for the treatment of HF. After years of study, we can obtain the reunderstanding of syndrome and formula syndrome in Chinese medicine [22]. Currently, Chinese herbal formulas are known to have an outstanding advantage with regard to bodily regulation [23]. Integrative medicine, an unprecedented task in present world, is a new pattern of medicine, which is formed by the integration of traditional Chinese medicine and Western medicine. In the past thirty years, great achievements have been made in integrative medicine researches, especially in clinical practice. Integrative medicine will do obtain greater achievements in creating new medicine and pharmacology [24].

WXKL is the first antiarrhythmic Chinese medicine to be approved by the state. It is the first drug that has effect on the multi-ion channel as proven by the patch-clamp technique. Through years of experimental research and clinical applications, WXKL has been proven to effectively treat arrhythmias [25]. WXKL was developed from the application of traditional medicine theory combined with the essence of Chinese and Western medicine theory. It is composed of five main components:* Nardostachys chinensis Batal* extract,* Codonopsis*, notoginseng, amber, and* rhizoma polygonati*. A study has investigated the influence of WXKL on the ventricular electrophysiology in rabbits with congestive heart failure (CHF). Compared with the HF group, the rabbits with HF treated by WXKL exhibited a significantly increased LVEF. The results showed that WXKL has protective function in heart failure accompanied by arrhythmia [26]. WXKL also has treatment effects on isoproterenol (ISO) induced heart failure in rats. It greatly improves the ISO induced cardiac dysfunction [27]. Animal experiments have indicated that Wenxin Granules can ameliorate cardiac function in rabbits with CHF [28, 29]. Our previous research indicated that WXKL affected the AP and blocked the *I*_Ca−L_, which ultimately resulted in the treatment of arrhythmias [30]. Nard extract, one of the main components in WXKL, has electrophysiological effects on the sodium channels and calcium channels in single rabbit ventricular myocytes. Nard extract has blocking effects on *I*_Na_ and *I*_Ca_ in a concentration dependent manner. Experimental results have shown that WXKL is a novel atrial elective sodium channel blocker and is effective in suppressing AF and preventing the induction of AF [31].

WXKL inhibits heart failure and cardiac arrhythmias through a mechanism that may involve the regulation of the CaMKII signal transduction pathway; this mechanism is similar to that of amiodarone. WXKL treatment can reduce the incidence of cardiac arrhythmias in a rat myocardial infarction model and increase the calcium transient amplitude in isolated cardiac myocytes from rats with myocardial infarctions [32]. The effect of the Wenxin particle on the electrophysiological characteristics of endocardium from the rat left ventricle to explore its mechanism on arrhythmias was examined. The results showed that the Wenxin particle might have an antiarrhythmic effect by prolonging the action potential duration (APD) and eliminating the triggered activities [33]. From the included articles, we can see WXKL has significant effect of improving the main symptoms such as palpitations, headache, insomnia, and dizziness. This meta-analysis showed that WXKL alone or combined with Western medicine demonstrated a potential effect of improving the LVEF and lowering BNP.

This systematic review has some limitations. Firstly, the quality of the methodology of the RCTs included in this systematic review was generally low. All of the included trials claimed randomization, but only one [10] of the studies reported the methodology used to generate the allocation sequence. The other trials mentioned only that “patients were randomized into two groups,” indicating potential selection bias. None of the trials mentioned the blinding of the participants and personnel, and the studies did not provide sufficient information for quality assessment. Five of the trials [9, 12, 16–18] were conducted by a single author, which could lead to performance bias. Only two trials [11, 13] reported dropouts or withdrawals, but without an intention-to-treat analysis. None of the trials provided a pretrial estimation of sample size, which could indicate a lack of statistical power to ensure an appropriate estimation of the therapeutic effect. It is well known that trials that are poorly designed methodologically show larger differences compared with rigorously conducted trials. All of the trials were conducted in China and published in Chinese, leading to publication bias. All of the RCTs claimed that the positive effect of WXKL combined with Western medicine is better than Western medicine alone or no medicine. While no negative findings were reported, we cannot eliminate the possibility of the unpublished material.

Secondly, the safety of Chinese herbal medicines is of general concern. Adverse effects were reported in five out of the eleven included trials [8, 12, 13, 16, 17]. Some adverse effects were not severe, and the patients spontaneously recovered without special treatments. Some adverse effects were irreversible. In total, the incidence of adverse reactions was lower in the experimental groups compared with the control groups. Six of the trials did not report any adverse effects. Due to the limited evidence provided by the eligible trials, conclusions about the safety of WXKL combined with Western medicine cannot be drawn from this study. In the future, larger-scale clinical trials with long-term follow-up are warranted to properly assess the safety of WXKL therapy.

Thirdly, publication and other biases may play an important role. We only identified and included trials published in Chinese and most of the trials are small samples with positive findings. We tried to avoid language bias and location bias, but we cannot exclude potential publication bias. While the articles were being assessed for eligibility, we found that the majority was excluded because there is no detailed information, and the quality of the methodology of the RCTs included in this systematic review is generally low. We recommend that future researchers should follow the basic guidelines for reporting clinical trials and conduct with a clearer TCM diagnostic criteria.

The currently available antiarrhythmic drugs for HF have limited safety and efficacy, most likely because they were not designed based on specific pathological mechanisms. We should understand the traditional and novel aspects of antiarrhythmic drugs in relation to the main pathological mechanisms of HF and present potential therapeutic approaches. We should continue to expand the cumulative meta-analysis of future trials, especially RCTs, and choose possible predictors of HF to provide more meaningful clinical indicators.

## 5. Conclusions

There is some encouraging evidence of WXKL, either alone or in combination with Western medicine, for improving the LVEF and BNP in patients with HF. After treatment with WXKL for eight weeks, the LVEF was significantly improved, and there was a significant reduction in the BNP levels in patients with HF. Because of the poor quality of the experimental design and methodology, the evidence for WXKL remains weak. More rigorous RCTs with strong designs and high methodological quality will be needed to present a high level of evidence for the effectiveness of WXKL in treating HF.

## Figures and Tables

**Table 1:** Characteristics and methodological quality of the included studies.

Study	Sample size (*T*/*C*)	Diagnosis standards	Complications	Intervention	Control	Treatment course (week)	Clinical standards	Outcome measure
Wang et al. 2009 [8]	77 (40/37)	Diagnostic criteria for CHF (unclear)	CHF	WXKL 9 g tid + Control	Metoprolol	8	Unclear	LVEF, ADR
Weng 2012 [9]	168 (88/80)	Diagnostic criteria for CHF (unclear)	CHF	WXKL 9 g tid	Conventional therapy (no detailed information)	8	Unclear	LVEF
Yu et al. 2006 [10]	72 (37/35)	Guiding principle of TCM treatment of CHF (1993)	CHF	WXKL 6 g tid	Conventional therapy (no detailed information)	8	Guiding principle of clinical research on new drugs of TCM (1993)	LVEF
Jia et al. 2011 [11]	42 (21/21)	Diagnostic criteria for CHF (unclear)	CHF and PAF	WXKL 9 g tid	Conventional therapy (no detailed information)	8	Unclear	LVEF
Jin 2010 [12]	60 (30/30)	Diagnostic criteria for CHF (unclear)	CHF and VPB	WXKL 9 g tid	Conventional therapy (no detailed information)	8	Unclear	LVEF, BNP, ADR
Yue et al. 2012 [13]	100 (50/50)	Chinese diagnosis and treatment of CHF guidelines (2007)	CHF and PAF	WXKL 9 g tid	Conventional therapy (no detailed information)	8	Chinese diagnosis and treatment of CHF guidelines (2007)	LVEF, BNP, ADR
Kong et al. 2009 [14]	60 (30/30)	Diagnostic criteria for CHF (unclear)	CHF	WXKL 9 g tid	Conventional therapy (no detailed information)	8	Unclear guiding	LVEF, BNP
Yang and Dong 2009 [15]	80 (40/40)	Diagnostic criteria for CHF (unclear) Chinese diagnosis	CHF	WXKL 9 g tid	Conventional therapy (no detailed information)	8	Principle of clinical research on new drugs of TCM	LVEF, BNP
Hu 2010 [16]	98 (50/48)	Chinese diagnosis and treatment of CHF guidelines (2007)	CHF	WXKL 9 g tid	Conventional therapy (no detailed information)	8	Unclear	LVEF, BNP, ADR
Wang 2012 [17]	70 (35/35)	ACC/AHA	CHF	WXKL 9 g tid	Conventional therapy (no detailed information)	8	New drug Clinical guidelines (Unclear)	LVEF, BNP, ADR
Xu 2012 [18]	80 (48/32)	Chinese diagnosis and treatment of CHF guidelines (2007)	CHF	WXKL 9 g tid	Conventional therapy (no detailed information)	8	Unclear	LVEF, BNP

**Table 2:** Quality assessment of the included randomized controlled trials.

Included trials	Sequence generation	Allocation concealment	Blinding of participants personnel	Blinding of outcome assessors	Incomplete outcome data	Selective outcome reporting	Other sources of bias	Risk of bias
Wang et al. 2009 [8]	Unclear	Unclear	Unclear	Unclear	Unclear	No	Unclear	High
Weng 2012 [9]	Unclear	Unclear	Unclear	Unclear	Unclear	No	Unclear	High
Yu et al. 2006 [10]	Table of random number	Unclear	Unclear	Unclear	Unclear	No	Unclear	Unclear
Jia et al. 2011 [11]	Unclear	Unclear	Unclear	Unclear	Yes	No	Unclear	High
Jin 2010 [12]	Unclear	Unclear	Unclear	Unclear	No	No	Unclear	High
Yue et al. 2012 [13]	Unclear	Unclear	Unclear	Unclear	Yes	No	Unclear	High
Kong et al. 2009 [14]	Unclear	Unclear	Unclear	Unclear	Unclear	No	Unclear	High
Yang and Dong 2009 [15]	Unclear	Unclear	Unclear	Unclear	Unclear	No	Unclear	High
Hu 2010 [16]	Unclear	Unclear	Unclear	Unclear	Unclear	No	Unclear	High
Wang 2012 [17]	Unclear	Unclear	Unclear	Unclear	Unclear	No	Unclear	High
Xu 2012 [18]	Unclear	Unclear	Unclear	Unclear	Unclear	No	Unclear	High
